# Plasmon Resonances in 1D Nanowire Arrays and 3D Nanowire Networks of Topological Insulators and Metals

**DOI:** 10.3390/nano13010154

**Published:** 2022-12-29

**Authors:** Olga Caballero-Calero, Alejandra Ruiz-Clavijo, Cristina V. Manzano, Marisol Martín-González, Gaspar Armelles

**Affiliations:** Instituto de Micro y Nanotecnología, IMN-CNM, CSIC (CEI UAM+CSIC) Isaac Newton, 8, Tres Cantos, E-28760 Madrid, Spain

**Keywords:** plasmon resonances, thermoelectric materials, topological insulators

## Abstract

The 1D nanowire arrays and 3D nanowire networks of topological insulators and metals have been fabricated by template-assisted deposition of Bi_2_Te_3_ and Ni inside anodic aluminum oxide (AAO) templates, respectively. Despite the different origins of the plasmon capabilities of the two materials, the results indicate that the optical response is determined by plasmon resonances, whose position depends on the nanowire interactions and material properties. Due to the thermoelectric properties of Bi_2_Te_3_ nanowires, these plasmon resonances could be used to develop new ways of enhancing thermal gradients and their associated thermoelectric power.

## 1. Introduction

**Figure 1:**
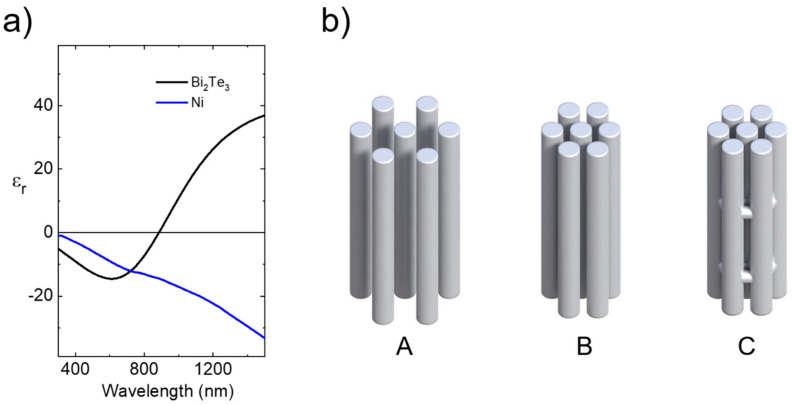
(**a**) Spectral dependence of the real part of *ε_//_*$\left({\mathrm{Bi}}_{2}{\mathrm{Te}}_{3}\right)$ and that of Ni, experimentally obtained in this work; (**b**) schemes of the different nanowire arrays, being **A**: 1D with a larger inter-wire distance, **B**: 1D with a shorter inter-wire distance, and **C**: 3D with inter-wire connections and the same inter-wire distance as B.

In the last few years, both the theory and experiments of plasmonic research have experienced a strong evolution. This has made the realization of photonic devices with a variety of customizable features possible [1,2,3]. One limiting factor in the development of this field has been energy dissipation. Most of the metals normally used in plasmonics suffer from losses, particularly in the visible and ultraviolet (UV) spectral ranges. This has spurred research into alternate low-loss plasmonic materials [4,5]. The A_2_B_3_ chalcogenide family (where A = Bi, Sb, and B = S, Se, Te) appears promising in this regard [6]. This family has been extensively studied as thermoelectric materials, being among the most efficient in transforming thermal gradients into electrical voltage around room temperature [7,8]. Their crystal structure consists of a sequence of five layers of A and B atoms arranged into a hexagonal lattice cell. They have a diagonal dielectric tensor with in-plane (*ε*_//_, perpendicular to the vertical *c*-axis) and out-of-plane (*ε_⊥_* parallel to the vertical *c*-axis) dielectric constants, respectively [9,10]. Moreover, they exhibit strong spin-orbit coupling, which generates highly conducting surface states and a topological insulator (TI) behavior [11,12]. On the one hand, these surface states have distinct conduction channels for spin-up and spin-down electrons, which prevent back-scattering and allow robust plasmon resonances, mainly localized in the mid- and far-infrared spectral ranges [13,14,15,16,17,18]. On the other hand, inter-band transitions are responsible for the visible and UV optical responses [6,19,20] with spectral regions where the real part of the dielectric constant has negative values (see Figure 1a). In these spectral regions, these compounds may present plasmonic behavior, and several works have shown plasmon resonances in the visible and UV spectral ranges in nanostructures made of them [21,22,23,24,25]. Moreover, due to the high negative values of the real part of the dielectric constant, they have been proposed as alternative materials for the development of UV devices based on UV plasmonics [6], and for infrared photonic applications [26]. In these chalcogenides, the plasmonic behavior originates from a different mechanism than that of standard plasmonic materials, such as Drude-like metals, such as Ni (see Figure 1a). Contrary to the preceding case, Ni has a negative value in the whole spectral range, and, therefore, when embedded in a dielectric medium, the resulting nanocomposite may be able to support plasmon resonances in a much wider spectral range [27]. The dielectric medium in our case is the porous anodic aluminum oxide (AAO) membranes, which have been extensively used as templates to fabricate inside their nanopores different types of nanostructures, such as nanowires or nanotubes [28]. Most of the works that can be found in the literature deal with 1D nanoporous membranes, but in 2014, a new method was proposed to fabricate 3D nanoporous membranes [29]. In these structures, the vertical pores of 1D alumina membranes are connected to the surrounding pores by a network of horizontal tubes, resulting in a 3D mesh of pores. In this work, we have used both the standard 1D and the novel 3D AAO templates to fabricate both 1D and 3D nanostructures of Bi_2_Te_3_ and analyze the plasmonic behavior of these TI nanowire arrangements. This is done by analyzing the optical properties of two sets of nanowire arrays with very similar wire diameter, but different inter-wire distance in the case of 1D nanowire arrays (A and B in Figure 1b), or with the presence of inter-wire connections when compared with 3D nanowire networks (C in Figure 1b). By reducing the inter-wire distance, the interaction between the nanowires is increased, and it can be further increased by connecting the wires between them. The ease and cost-effectiveness of the fabrication method (based on anodization and electrochemical deposition) used for both the 1D nanowire arrays and 3D nanowire networks, along with the possibility of changing the geometrical parameters of the nanostructures, is an advantage when compared with other more complicated techniques of obtaining nanostructures, such as two-photon optical lithography [30,31] for template fabrication or atomic layer deposition (ALD) for the fabrication of the nanostructured materials [32]. We will analyze how this modification of the interaction affects the plasmonic behavior of the entire nanostructure and compare this behavior with identical 1D nanowire arrays and 3D nanowire networks made of a metal, such as Ni, which, as can be seen in Figure 1a, has a different capability of supporting plasmon resonances. 

## 2. Materials and Methods

### 2.1. Fabrication of the 1D Nanowire Arrays and 3D Nanowire Networks

The nanowire arrays are fabricated by template-assisted deposition of the different materials (Bi_2_Te_3_ and Ni) inside anodic aluminum oxide (AAO) templates, which were also fabricated for this work. The process for obtaining 1D and 3D nanowire arrays has been thoroughly described in previous works from our group (see, for instance, [28,33,34,35,36]), but it can be explained as follows. The AAO templates are fabricated by a two-step anodization process on an aluminum substrate, with the same first step for all the templates used in this work and a second anodization step that varies depending on the kind of template, that is, 1D-AAOs with a 50 nm pore diameter and distances between the pores of 65 or 100 nm or 3D-AAOs. In the case of the templates with 50 nm of pore diameter and 100 nm of inter-pore distance, both anodization processes are carried out in 0.3 M oxalic acid (H_2_C_2_O_4_) under 40 V at 3 °C for 24 h. In all cases, after the first anodization, the anodic alumina is etched. This produces an ordered pattern in the aluminum substrate that is used as an ordered template for the second anodization process. After removing the remaining aluminum with an aqueous solution of HCl/CuCl_2_ and the barrier layer with a 10 wt.% H_3_PO_4_ solution at 30 °C, the final templates have a 50 nm pore diameter and a 100 nm inter-pore (inter-wire) distance (as shown in the scheme of A in Figure 1b). 

B and C in Figure 1b show 1D-AAOs with a 65 nm inter-pore distance and 3D-AAOs, respectively; the first anodization is carried out in 0.3 M sulfuric acid (H_2_SO_4_) under 25 V at 0 °C for 24 h, followed by the etching step described before. Then, the second anodization at a constant voltage is performed in the case of 1D-AAOs, and at pulsed voltage alternating between mild and hard anodization for the 3D-AAOs. In both cases, after removing the aluminum and the barrier layer with the same process as in the case of oxalic templates, a final etching treatment with 5 wt.% H_3_PO_4_ was carried out. In the case of 3D-AAOs, this final process opens the transversal channels that connect the structures. The final 3D-AAO structure consists of pores of 50 nm in diameter and 65 nm of inter-pore distance, connected along a certain length (which is controlled by the second anodization pulses) to their first neighbors by transversal channels of around 30 nm in diameter (see C in Figure 1b). In the case of 1D-AAOs, this etching step is needed to obtain a final diameter of the pores of 50 nm (Figure 1b). More details on the template fabrication can be found in references [28,29,37,38].

The nanostructures were grown by electrodeposition using a three-electrode electrochemical cell. The anodic alumina templates were evaporated with 5 nm Cr and 150 nm Au on one side and glued with silver paint to a copper electrode, which is used as the working electrode, after covering the copper with nail polish to leave only the surface of the template exposed to the electrolyte. The other two electrodes are an Ag/AgCl as reference electrode and a platinum mesh as counter electrode. The electrolyte solution for the fabrication of Bi_2_Te_3_ nanostructures contains 0.9 × 10^−2^ M Bi^3+^ (from Aldrich® bismuth pieces 99.999%), 10^−2^ M HTeO_2_^+^ (from Aldrich® tellurium powder 99.997%), and 1 M HNO_3_ (from Panreac® 65%), and the electrodepositions were performed at a controlled temperature of 0 °C. In the case of the Ni nanostructures fabrication, the solution consists of 0.75 M NiSO_4_·6H_2_O, 0.02 M NiCl_2_·6H_2_O, 0.4 M H_3_BO_3_, and 0.016 M of saccharine, and the process was performed at 45 °C. The growth was carried out by pulsed electrodeposition between an applied potential of 0.04 V vs. Ag/AgCl and zero density current in the case of Bi_2_Te_3_ and at constant potential of −0.9 V vs. Ag/AgCl in the case of Ni. These processes were optimized in previous works of our group, [39] and [36], respectively.

Then, the nanostructures were polished with aluminum powder of 5, 3, and 0.1 microns in diameter to remove the gold and chromium layers and have access to the nanostructured material underneath to perform the optical characterizations.

### 2.2. Characterization of the 1D Nanowire Arrays and 3D Nanowire Networks

The morphological characterization of the nanostructures was carried out with a high-resolution scanning electron microscope (HR-SEM, FEI Verios 460), and the crystal structure and orientation were measured with two pieces of equipment, an XRD Philips X’Pert four circles diffractometer and a D8 Discovery X-ray diffractometer (from Bruker), both with a CuKα X-ray transmitter. The spectroscopic ellipsometry was performed with an M2000 FI from J. A. Woollam Co. The nanostructures were characterized in the spectral range from 300 nm to 1500 nm. In this technique, the ratio between the reflectivity of p and s polarized light was obtained at different incident angles (55° to 75° in steps of 10 degrees). The analysis of the results was performed with the software Complete EASE and WVase32.

## 3. Results and Discussion

### 3.1. Morphological and Structural Characterization of the 1D Nanowire Arrays and 3D Nanowire Networks

**Figure 2:**
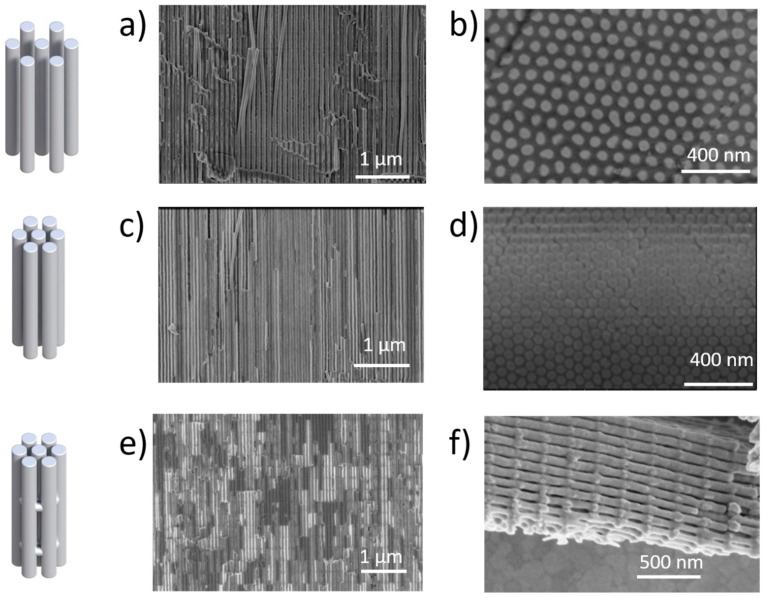
Scanning electron microscopy images of the different nanostructures fabricated with Bi_2_Te_3_, in side view (**a**,**c**,**e**) and top view (**b**,**d**) before dissolving the alumina template. The schemes of the different structures (as shown in Figure 1) are displaced on the left hand of the figure in the same row as their SEM counterparts. (**a**,**b**) correspond to the 1D nanowire array with the nanowire inter-distance of 100 nm (side and top views, respectively), (**c**,**d**) correspond to the 1D nanowire array with nanowire inter-distance of 65 nm (side and top views, respectively), and (**e**,**f**) correspond to the 3D nanowire network, (**e**) being the side view inside the 3D-AAO template and (**f**) after dissolving the 3D-AAO template.

**Figure 3:**
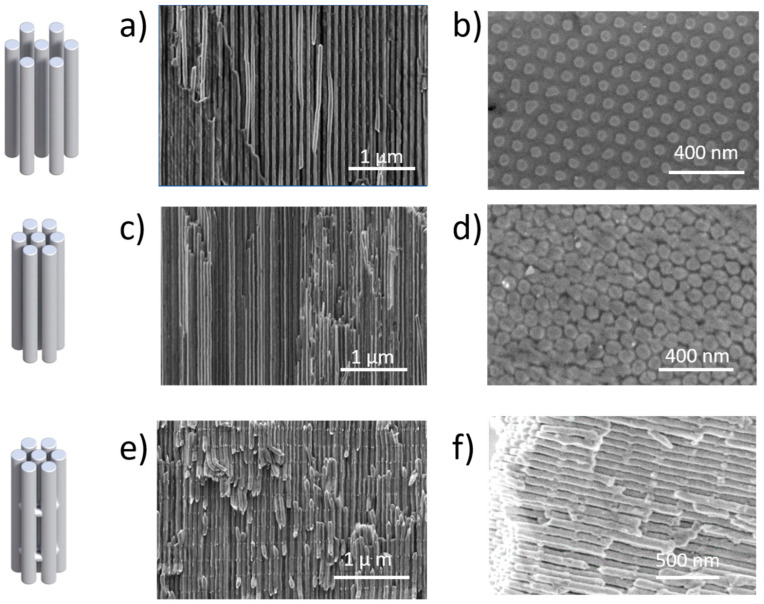
Scanning electron microscopy images of the different nanostructures fabricated with Ni, along with the schemes shown in Figure 1 (displayed in the left hand of the figure) corresponding to the right SEM images. (**a**,**b**) correspond to the 1D nanowire array with the nanowire inter-distance of 100 nm (side and top views, respectively), before dissolving the AAO template, (**c**,**d**) correspond to the 1D nanowire array with 65 nm of inter-wire distance (side and top views, respectively), and (**e**,**f**) correspond to the 3D nanowire network, inside the alumina template and self-standing, once the 3D-AAO has been dissolved, respectively.

The diameters of the nanowires used in this work have been sized at 50 ± 5 nm. There were two kinds of 1D nanowire arrays, with 100 or 65 nm of inter-wire distance (corresponding to the schemes of A and B in Figure 1b, respectively). Scanning electron images of those nanostructures can be found in Figure 2 for Bi_2_Te_3_ and Figure 3 for Ni. In the case of the 3D nanowire networks, the longitudinal nanowires also have 50 nm pore diameter and 65 nm inter-wire distance. Then, the connections between neighboring nanowires have diameters of 30 ± 5 nm and are separated by 220 ± 10 nm along the nanowire length.

**Figure A1:**
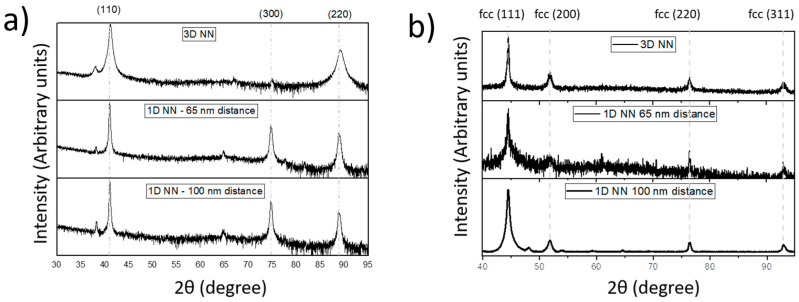
X-ray diffraction measurements of the different 1D and 3D nanowire structures made of (**a**) Bi_2_Te_3_ and (**b**) Ni. The different peaks have been identified with the references JCPDS-ICDD-00-0150863 in the case of Bismuth Telluride and JCPDS-ICDD-00-0040850 for Nickel.

The crystal orientation of the nanostructures was analyzed through X-ray diffraction (XRD) measurements, which are shown in Figure A1. In the case of the different nanostructures of Bi_2_Te_3_ (Figure A1a), it is clearly shown that they present a preferential orientation along the [110] direction, which means that the *c*-axis is oriented perpendicular to the length of the nanowire. Therefore, the bismuth and tellurium planes of the Bi_2_Te_3_ structure are aligned parallel to the long direction of the nanowires. In the case of nickel (Figure A1b), the diffraction pattern obtained for the three different nanostructures show only peaks corresponding to the face-centered cubic (FCC) structure, demonstrating that it is a polycrystalline material. It is worth mentioning here that these kinds of nanostructures have already been fabricated and measured in previous works of our group (see, for instance, [33,35,36,39,40]), which has the expertise of controlling the growth parameters in such a way that the nanostructures obtained are highly reproducible.

### 3.2. Optical Characterization of the 1D Nanowire Arrays and 3D Nanowire Networks

The ellipsometry data were obtained for the range 300 to 1500 nm, at incident angles of 55, 65, and 75 degrees with respect to the normal to the surface of the sample. In the different nanostructures, no dependence of the ratio between the reflectivity of *p* and *s* polarized light with the in-plane orientation of the sample was observed, as expected from X-ray diffraction characterization. Moreover, the nanowire layers are thick enough to be optically opaque, and due to the nanowire structure, they should be considered as uniaxial with in-plane and out-of-plane optical properties. Furthermore, as the sizes of the nanowires and inter-nanowire distances are much smaller than the wavelength, the nanowire structures can be viewed as an effective medium, and the ellipsometry measurements can be analyzed in terms of an effective dielectric tensor with in-plane (perpendicular to the nanowire axis) and out-of-plane (parallel to the nanowire axis) effective dielectric constants obtained from the ellipsometry data.

**Figure 4:**
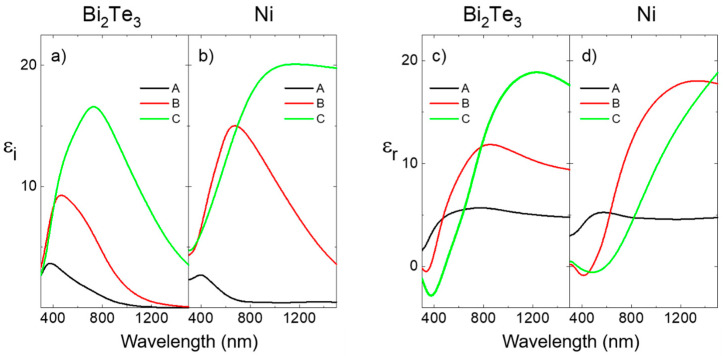
Experimental spectra of the imaginary part (**a**,**b**) and real part (**c**,**d**) of the in-plane effective dielectric constant of 1D nanowire arrays with 100 nm inter-wire distance (black line, labeled A), 1D nanowire arrays with 65 nm inter-wire distance (red line, labeled B) and 3D nanowire network (green curve, labeled C) made of Bi_2_Te_3._ (**a**,**c**) and Ni (**b**,**d**).

The imaginary and real parts of the in-plane effective dielectric constant obtained for the different structures assuming a sharp air/layer interface are shown in Figure 4a–d. We would like to point out that due to the values of the in-plane effective constants, the sensitivity of the ellipsometry data to the out-of-plane dielectric constant is very small and will not be discussed here. 

As can be observed for both the Bi_2_Te_3_ (Figure 4a) and Ni (Figure 4b) 1D nanowire arrays (black and red curves), the imaginary part of the in-plane dielectric constant shows a peak whose position and structure depend on the material and inter-wire distance. For Bi_2_Te_3_ structures, the dielectric constant shows a peak and a broad shoulder at the low energy side. As the inter-wire distance decreases, the intensity of the peak and the shoulder increase, and their positions shift towards lower energies. In the same way, Ni 1D nanowire arrays show a peak whose position and intensity have the same evolution with the inter-wire distance as that of 1D Bi_2_Te_3_ structures. These peaks are due to the plasmon resonances of the wires, whose positions and intensities depend on the inter-wire distance and material properties. On the one hand, the real part of the effective dielectric constant shows an s-like structure at the position of the resonances, whose evolution with the inter-wire distance and material follows the same trend as the imaginary part. On the other hand, the shape of the spectra of the 3D nanowire networks (green curves) is roughly very similar to the 1D nanostructures but shifted to lower energies. This redshift is higher for Ni than for Bi_2_Te_3_. Furthermore, the Ni feature is much broader than the Bi_2_Te_3_ one.

**Figure 5:**
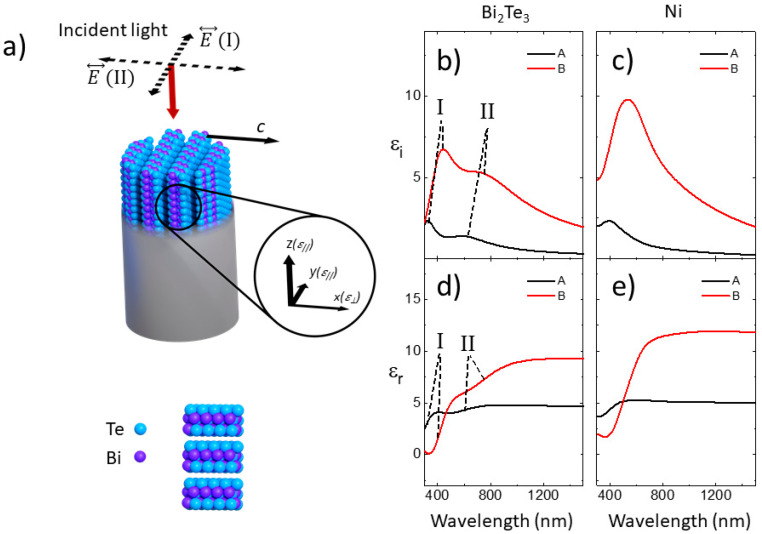
(**a**) Scheme of the relative orientation of Bi_2_Te_3_ dielectric constants deposited inside the alumina templates, with the *c*-axis perpendicular to the nanowire axis. The bismuth and tellurium atoms are represented schematically by violet and blue spheres, respectively. A scheme of the different polarization of the incident light, which excite plasmon resonances, E(I) (polarization perpendicular to the *c*-axis) and E(II) (polarization parallel to the *c*-axis), is shown. These components are responsible for the plasmons labeled I and II. (**b**–**e**) Calculated spectra of the imaginary part (**b**,**c**) and real part (**d**,**e**) of the in-plane effective dielectric constant of 1D nanowire arrays with 100 nm inter-wire distance (black line, labeled A), and 1D nanowire arrays with 65 nm inter-wire distance (red line, labeled B) made of Bi_2_Te_3_ (**b**,**d**) and Ni (**c**,**e**).

As has already been discussed, the 1D nanowire arrays of both materials show similar optical properties and a similar evolution with the inter-wire distance, but there are some differences related to the different nature of the wire material. On the one hand, the spectral shape of the Bi_2_Te_3_ features is linked to the growth conditions of the Bi_2_Te_3_ arrays, which result in nanowire arrays with the *c*-axis perpendicular to the wire axis, as shown in the XRD of Figure A1. Therefore, the optical properties of the nanowires will depend on the relative orientation of the light polarization and the *c*-axis, giving rise to two main resonances: one for light polarized along this axis and the other in the perpendicular direction (which is the direction along which the bismuth and tellurium are aligned, see Figure 5a). On the other hand, due to the cubic structure of Ni, only one resonance is observed for Ni 1D nanowire arrays. 

To clarify the origin of those plasmon resonances, we have modeled the nanowire structure using a Maxwell Garnet (MG) approximation and taking into account the uniaxial structure of Bi_2_Te_3,_ which, due to the deposition conditions, has the hexagonal *c*-axis oriented perpendicular to the wire axis (see Figure 5a). Figure 5b–e show the calculated dielectric constants for the 1D nanowire arrays. The random in-plane orientation of the *c*-axis has been taken into account, as explained in Appendix B. These curves allow a clear identification of the different features of the experimental result. For Bi_2_Te_3_ nanowire arrays, the main peak of the imaginary part and the *s*-like structure of the real part located at higher energy (labeled Ι in Figure 5b,d) correspond to the excitation of the plasmon resonance of the wires that have the *c*-axis perpendicular to the light polarization (see Figure 5a), whereas the shoulders of both the imaginary and real part (labeled II in Figure 5b,d) result from the excitation of the plasmon resonance of the wires with the *c*-axis parallel to the light polarization (see Figure 5a). As the distance between the nanowires is reduced, the interaction between them increases, and as a result, the position of both resonances shifts towards lower energies, as experimentally observed. In the same figure, the calculated spectra for Ni 1D nanowire arrays is also shown. Contrary to the preceding example, for all in-plane polarizations of the light, only one plasmon resonance is excited, which gives rise to the peak of the imaginary part and the s-like feature of the real part, whose position shifts towards lower energies as the inter-wire distance decreases due to the increase in the interaction between the nanowires.

These results support the identification of the different features as due to the excitation of plasmon resonances in the nanostructures and give some clues about the different evolution of the optical properties of the two materials under study, Bi_2_Te_3_ and Ni, when the nanowires are physically interconnected (forming the 3D nanowire networks). On the one hand, in the case of Ni, which may support plasmon resonances in the whole spectral range, the connection between the nanowires increases the interaction between the plasmons, inducing an additional redshift of the resonance. On the other hand, for Bi_2_Te_3_, this additional shift is reduced due to the restriction of the spectral range where plasmon resonances occur. A very rough estimation of the material dependence of this additional shift could be obtained using the same Maxwell Garnett approximation, and considering that the in-plane connection of the nanowires can be viewed as a change of the shape of the nanostructure from a nanowire to an effective shape defined by an effective form factor. This effective form factor should be the same for the two materials and can be determined from the redshift of the Ni nanostructures, which amounts to 490 nm, whereas within this approximation the estimated Bi_2_Te_3_ redshift is 220 nm, very similar to the experimental one, which is 260 nm.

The comparison between simulated and experimental results is good, and the origin of the slightly different result for Bi_2_Te_3_ is not fully understood. It might be due to the different optical properties of the Bi_2_Te_3_ grown inside the nanopore as compared with that grown as films, as it has also been observed for other chalcogenide compounds whose optical properties depend on the deposition conditions [41]. Moreover, due to the strong interface localization of the plasmon resonances, the interface between the alumina matrix and the wire plays an important role in determining both the position and intensity of the resonances. In the calculated spectra, we have assumed sharp interfaces between the alumina matrix and the wire material, which could not be the case for the experimental structure and may also depend on the nature of the wire material.

## 4. Conclusions

In summary, in this work, 1D nanowire arrays and 3D nanowire networks made of topological insulators and metals have been fabricated using 1D and 3D alumina templates. Their optical properties have been analyzed using spectroscopic ellipsometry. Despite the different origins of the plasmon capabilities of the two materials, the results indicate that the optical response is determined by plasmon resonances, whose position depends on the nanowire interactions and material properties. Furthermore, given that Bi_2_Te_3_ 3D nanowire networks outperform their bulk or thin film counterparts regarding their thermoelectric efficiency [40] and that these metamaterials can be used to fabricate miniaturized thermoelectric generators, where establishing a thermal gradient can be challenging, the possibility of obtaining an additional localized temperature increase by using these plasmon resonances would result in an increase in the final power output. In our case, the light confinement produced by the plasmonic resonance in the Bi_2_Te_2_ 1D and 3D nanostructures would increase the effective thermal gradient in the material, and thus, increase the thermoelectric power output obtained in the nanostructured material. These metamaterials should help to develop plasmon-assisted thermoelectric devices similar to the resonant thermoelectric nanophotonic devices recently proposed [42].

## Figures and Tables

**Figure A2:**
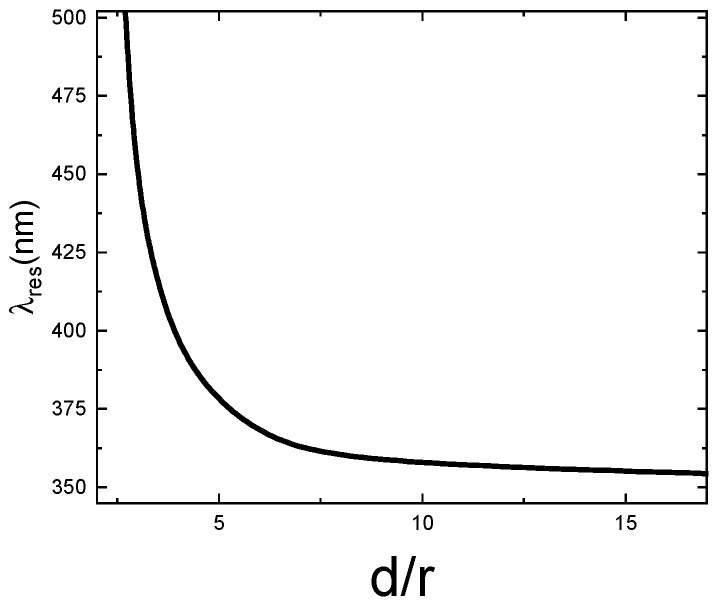
Dependence of the position of the peak of the imaginary part of the in-plane effective dielectric tensor for the case of Ni nanowire arrays as a function of the distance between the nanowires.

**Figure A3:**
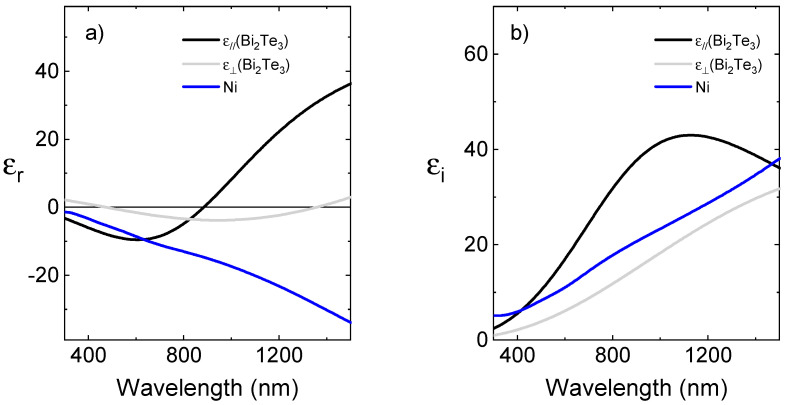
Experimentally obtained (**a**) real and (**b**) imaginary optical constants of Bi_2_Te_3_ (black and gray lines) and Ni (blue line).

## Data Availability

Not applicable.

## Appendix B. Details of the Optical Model

The wire layers have been modeled using a Maxwell Garnett approximation [43,44]. Within this approximation, the dielectric constant of the Ni wire arrays can be related to the dielectric constants of Ni and alumina as follows:

(1) $$\varepsilon_{xx,yy}=\frac{\left[\left(1+f\right)\varepsilon \left(Ni\right)+\left(1-f\right)\varepsilon \left(Al_{2}O_{3}\right)\right]\varepsilon \left(Al_{2}O_{3}\right)}{\left(1-f\right)\varepsilon \left(Ni\right)+\left(1+f\right)\varepsilon \left(Al_{2}O_{3}\right)}$$

(2) $$\varepsilon_{z}=f\varepsilon \left(Ni\right)+\left(1-f\right)\varepsilon \left(Al_{2}O_{3}\right)$$

where $f=\frac{\pi }{\sin60}{\left(\frac{r}{d}\right)}^{2}$, r being the radius of the wire and d the inter-wire distance,  ε (Ni) and $\varepsilon \left(Al_{2}O_{3}\right)$ are the Ni and alumina dielectric constants, respectively.

For Bi_2_Te_3_ wires arrays, however, we must consider the uniaxial character of Bi_2_Te_3_, and thus, for the nanowires arrays with the Bi_2_Te_3_ *c*-axis parallel to the *x*-axis, the diagonal components of the effective dielectric tensor of the wire layers can be related to the Bi_2_Te_3_ dielectric constant parallel and perpendicular to the *c*-axis, as follows:

(3) $$\varepsilon_{xx}\left(\theta =0\right)=\frac{\left[\left(1+f\right)\varepsilon_{⊥}\left(Bi_{2}Te_{3}\right)+\left(1-f\right)\varepsilon \left(Al_{2}O_{3}\right)\right]\varepsilon \left(Al_{2}O_{3}\right)}{\left(1-f\right)\varepsilon_{⊥}\left(Bi_{2}Te_{3}\right)+\left(1+f\right)\varepsilon \left(Al_{2}O_{3}\right)}$$

(4) $$\varepsilon_{yy}\left(\theta =0\right)=\frac{\left[\left(1+f\right)\varepsilon_{//}\left(Bi_{2}Te_{3}\right)+\left(1-f\right)\varepsilon \left(Al_{2}O_{3}\right)\right]\varepsilon \left(Al_{2}O_{3}\right)}{\left(1-f\right)\varepsilon_{//}\left(Bi_{2}Te_{3}\right)+\left(1+f\right)\varepsilon \left(Al_{2}O_{3}\right)}$$

(5) $$\varepsilon_{zz}\left(\theta =0\right)=f\varepsilon_{//}\left(Bi_{2}Te_{3}\right)+\left(1-f\right)\varepsilon \left(Al_{2}O_{3}\right)$$

where *θ* is the angle between the *x* and *c*-axis, $\varepsilon_{⊥}\left(Bi_{2}Te_{3}\right)$ and $\varepsilon_{//}\left(Bi_{2}Te_{3}\right)\;$ are the $Bi_{2}Te_{3}$ dielectric constants parallel and perpendicular to the *c*-axis, respectively, and $\varepsilon \left(Al_{2}O_{3}\right)$ the alumina dielectric constant. The in-plane random orientation of the *c*-axis has been taken into account by rotating the dielectric tensor, $\overset{=}{\varepsilon \left(\theta_{i}\right)}$, an angle *θ_i_* around the axis perpendicular to the layer (*z*-axis) and taking the average value of all the dielectric tensors: $\langle \overset{=}{\varepsilon }\rangle =\frac{1}{n}\overset{n}{\underset{i=1}{\sum }}\overset{=}{\varepsilon \left(\theta_{i}\right)}$. For the sake of simplicity, the average value has been taken for *θ* = 0, 45, 90 and 135° dielectric tensors.

We would like to point out that the in-plane effective dielectric constant of the wire array can be rewritten in terms of the polarizability, *α*, of a single nanowire as:

(6) $$\varepsilon_{xx,yy}=\varepsilon \left(Al_{2}O_{3}\right)\left[\frac{1+f\alpha }{1-f\alpha }\right]$$

with $\;\alpha =\frac{\varepsilon \left(Wire\right)-\varepsilon \left(Al_{2}O_{3}\right)}{\varepsilon \left(Wire\right)+\varepsilon \left(Al_{2}O_{3}\right)}$. Therefore, the resonance of the polarizability, linked to the plasmon resonance of the nanowire, results in similar features in the effective dielectric constant and, in particular, it may give rise to peaks in the imaginary part of the dielectric constant, which can be identified as resulting from plasmon resonances of the wire arrays. Furthermore, as the inter-wire distance decreases, the interaction between them increases. This may result in changes in the position of the resonances, which can be analyzed using this effective medium approximation. As an example, we present in Figure A2, the dependence of the position of the peak of the imaginary part of the in-plane effective dielectric tensor for the Ni nanowires arrays as a function of the distance between the nanowires. As can be observed, when the nanowires are far apart, the resonance corresponds to that of the single nanowire (i.e., for diluted systems):

(7) $$\varepsilon_{xx,yy}\approx \varepsilon \left(Al_{2}O_{3}\right)\left[1+2f\alpha \right]$$

whereas, as the interaction increases, the resonance shifts towards lower energies, as it is experimentally observed.

Finally, the optical properties of the wire materials have been obtained from thick layers of Ni and Bi_2_Te_3_ grown in the same conditions used to fill the templates. (See Figure A3).
